# Biomarker-driven risk stratification and early intervention in acute kidney injury: a comprehensive review from early warning to clinical response

**DOI:** 10.3389/fmed.2026.1796756

**Published:** 2026-03-13

**Authors:** Kaihuan Zhou, Zhanhong Tang, Juntao Hu

**Affiliations:** Department of Critical Care Medicine, The First Affiliated Hospital of Guangxi Medical University, Nanning, China

**Keywords:** acute kidney injury, biomarkers, clinical decision support, early warning, risk stratification

## Abstract

Acute kidney injury (AKI) is a prevalent clinical syndrome in critically ill patients and is associated with adverse outcomes. Early detection has long relied on functional indicators, such as serum creatinine and urine output, which are inherently delayed. Recently, a growing body of biomarkers reflecting tubular structural injury, cellular stress and cell cycle arrest, inflammatory and immune activation, as well as metabolic and oxidative stress, has demonstrated utility in detecting subclinical kidney injury before overt functional deterioration. These biomarkers provide a novel biological basis for early AKI warning and risk stratification. Advances in continuous monitoring, time-series analysis, and artificial intelligence-based methods, the integration of multidimensional biological signals with dynamic clinical information has driven a paradigm shift in AKI early-warning research from static prediction toward dynamic risk assessment. This review synthesizes the mechanistic stratification of AKI biomarkers and their translational pathways within a closed-loop framework of early warning and clinical response. It focuses on integrated applications in Kidney Disease: Improving Global Outcomes-guided intervention strategies, individualized hemodynamic optimization, and multidisciplinary collaborative management. Furthermore, it analyzes challenges relating to standardized implementation, clinical heterogeneity, and real-world translation. The clinical value of AKI biomarkers extends beyond early risk identification; they function as triggers for structured clinical intervention pathways, facilitating a systematic linkage between risk assessment and therapeutic response, which promotes a transition of AKI management toward a mechanism-driven and prospective care paradigm.

## 1 Introduction

Acute kidney injury (AKI) is a clinical syndrome driven by multiple risk factors, characterized by complex pathophysiological mechanisms and marked heterogeneity. It is associated with high morbidity, mortality, and adverse long-term renal and cardiovascular outcomes (1, 2). Epidemiological studies indicate that the incidence of AKI among intensive care unit (ICU) patients ranges from 30 to 60%. In high-risk populations, such as those with sepsis, undergoing cardiac surgery, or experiencing multiple organ dysfunction, the risk is further amplified owing to the convergence of multiple pathological stresses (3–6). Although advances in critical care management and the application of renal replacement therapy (RRT) have improved short-term outcomes, overall mortality remains unacceptably high, and a substantial proportion of survivors develop chronic kidney dysfunction (1, 6).

The clinical diagnosis of AKI is largely based on functional indices, primarily serum creatinine and urine output. The development and refinement of the RIFLE, AKIN, and Kidney Disease: Improving Global Outcomes (KDIGO) criteria have significantly improved diagnostic consistency. However, these frameworks primarily focus on dynamic changes in renal function and fail to capture early structural injury and underlying pathological processes within the kidney (7–9). Serum creatinine, influenced by multiple factors, including age, muscle mass, volume status, and baseline renal function, and its elevation typically lags behind the onset of parenchymal kidney injury. Similarly, urine output is susceptible to confounding by fluid resuscitation, diuretic use, and hemodynamic conditions (10, 11). As a result, diagnostic frameworks for AKI relying on functional indicators have limited sensitivity for detecting injury at early or subclinical stages. Nevertheless, serum creatinine remains the globally recognized gold standard for AKI diagnosis, and its foundational role in clinical practice is unlikely to be replaced soon. However, this widely utilized marker does not reflect early biological processes of renal tissue injury, creating conflicts between the realities of current clinical practice and the ideal of an effective early warning tool.

Research has increasingly focused on early detection of structural injury and cellular stress. A series of molecular biomarkers reflecting tubular epithelial damage, cell cycle arrest, and inflammatory responses has been proposed, providing a novel biological basis for early AKI warning and risk stratification. Example, neutrophil gelatinase-associated lipocalin (NGAL) and kidney injury molecule-1 (KIM-1), which primarily indicate early tubular epithelial injury (12). The combination of tissue inhibitor of metalloproteinases-2 and insulin-like growth factor-binding protein 7 (TIMP-2·IGFBP7) reflects cellular stress and cell cycle arrest (13). Furthermore, interleukin-18 (IL-18) and liver-type fatty acid-binding protein (L-FABP) are closely linked to inflammatory responses and oxidative stress (14, 15).

Translating molecular biomarker signals into actionable clinical early warning tools remains a central challenge in AKI research. Advances in data integration capabilities and continuous monitoring technologies have increased attention to risk stratification models based on dynamic biomarker trajectories. This review summarizes recent advances in AKI biomarker research, specifically their roles in elucidating pathophysiological mechanisms, enabling early identification, and facilitating clinical translation, with the aim of constructing a multidimensional renal protection framework centered on “early warning and precision response.”

## 2 Literature search strategy and evidence integration

This structured narrative review aimed at integrating the mechanistic foundations, predictive value, and clinical translational significance of biomarkers for AKI. The literature search was performed in PubMed, the Web of Science Core Collection, and Embase, from January 1, 2000 to December 31, 2025. Particular emphasis was placed on studies published between 2020 and 2025, especially those from 2024 to 2025. This period reflects the most recent evidence base, characterized by the maturation of cell-cycle arrest biomarkers, the emergence of integrated proteomic and metabolomic profiling, and the increasing application of explainable artificial intelligence in AKI risk stratification.

**Figure 1:**
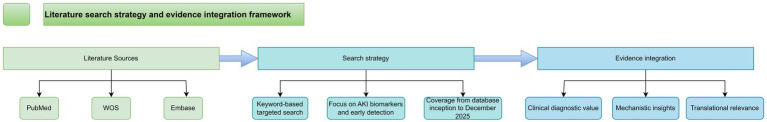
Literature search strategy and evidence integration framework. Relevant studies were identified through targeted searches of PubMed, Web of Science Core Collection, and Embase databases using keywords related to acute kidney injury biomarkers and early detection. The retrieved evidence was then integrated within a conceptual framework encompassing mechanistic insights, clinical diagnostic value, and translational relevance.

The search was performed using keywords, such as “acute kidney injury,” “biomarker,” “early detection,” along with representative molecular biomarkers. Studies were selected based on their representativeness and methodological quality related to pathophysiological mechanisms, predictive performance evaluation, or clinical translational applications. The included literature was then synthesized within a predefined conceptual framework using a structured narrative approach, highlighting the logical linkage between molecular injury signals and clinical risk stratification and response pathways (Figure 1).

## 3 Pathophysiological basis and biological logic of early warning

The core pathological process of AKI involves acute structural and functional disruption of renal tubular epithelial cells (1, 16). Ischemic, toxic, or inflammatory insults, cause rapid imbalances in tubular epithelial homeostasis, leading to necrosis or apoptosis (17, 18). Necrotic cells release damage-associated molecular patterns, which amplify inflammatory signaling through the Toll-like receptor-nuclear factor kappa B pathway and drive the progression of tissue injury.

In the early phase of structural damage, proximal tubular epithelial cells release molecules such as KIM-1 and NGAL (19–21). These biomarkers can be detected before increases in serum creatinine or reductions in urine output, making them sensitive indicators of subclinical AKI. A prospective study demonstrated that urinary NGAL levels were significantly higher in patients with acute tubular necrosis than in those with prerenal AKI and independently predicted short-term mortality (20).

As the pathological process progresses, injury extends from tubular epithelial cells to the microcirculation. Endothelial dysfunction leads to dysregulated vascular tone, reduced nitric oxide bioavailability, and enhanced vasoconstrictive signaling, thereby exacerbating renal medullary hypoxia (22, 23). During reperfusion, excessive generation of reactive oxygen species induces oxidative stress and microvascular injury, further impairing local perfusion and promoting progression toward irreversible AKI (24).

A reversible window exists in the early stage of AKI. During this phase, tubular epithelial cells undergo p53-mediated G1 cell cycle arrest, which limits the replication of damaged DNA and helps maintain metabolic homeostasis under sublethal stress (25). At this point, functional indicators may still remain within normal ranges, whereas molecular injury signals are already detectable, providing a basis for identifying subclinical AKI and enabling early intervention.

The pathological trajectory of AKI evolves from structural injury to functional failure, during which various biomarkers correspond to distinct dynamic stages of this process. KIM-1 and NGAL indicate structural damage, while TIMP-2·IGFBP7 reflects cell cycle arrest, and IL-18 and L-FABP represent inflammatory and oxidative stress responses (14, 19). Integrating these signals facilitates the early detection of kidney injury before abnormalities in serum creatinine or urine output become apparent.

## 4 Mechanism-linked biomarkers and predictive pathways

**Figure 2:**
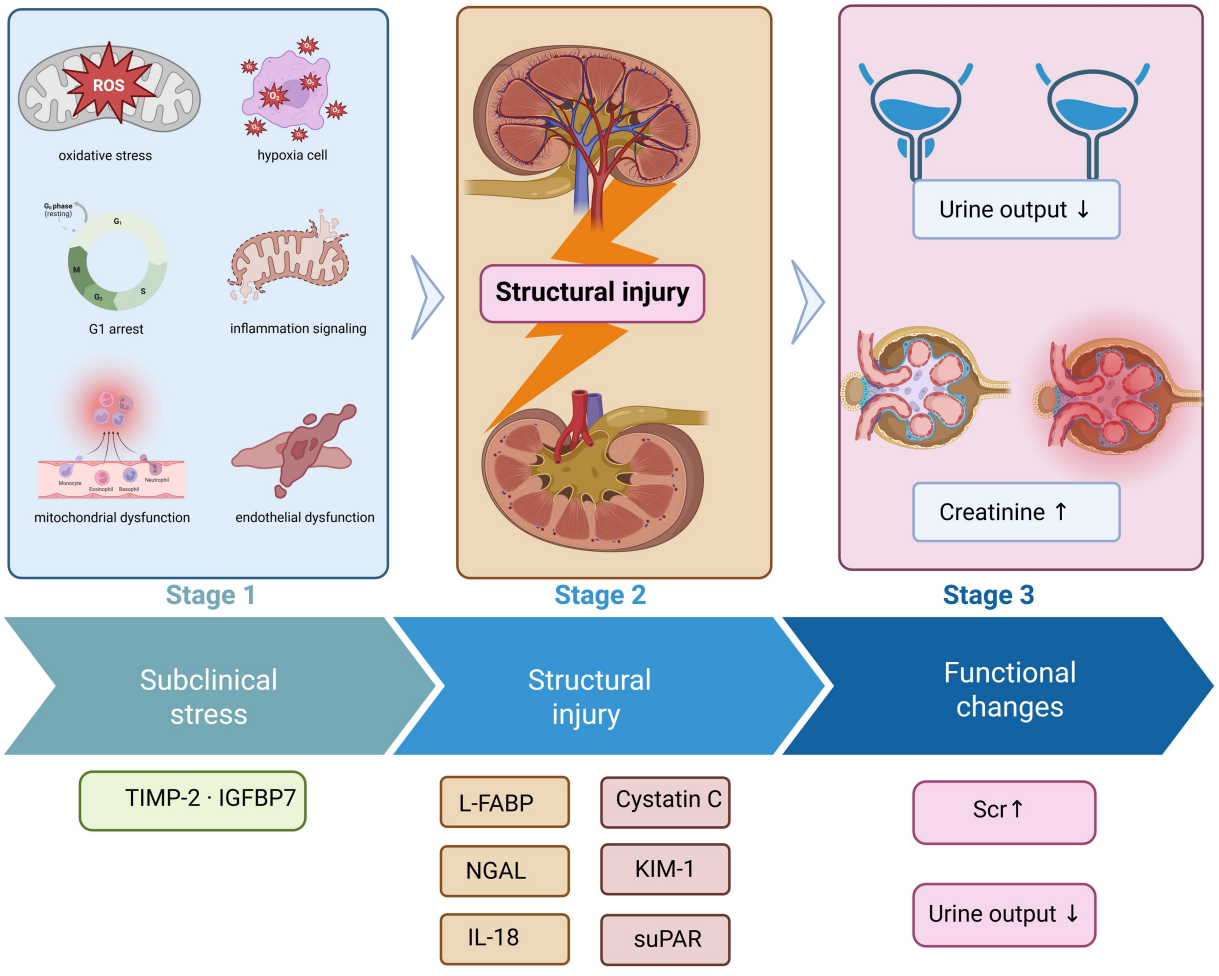
Biological timeline of acute kidney injury and stage-specific biomarker emergence. This schematic illustrates the temporal progression of acute kidney injury from early subclinical cellular stress, through overt structural injury, to subsequent functional decline. During the initial subclinical phase, renal tubular epithelial cells undergo cellular stress responses, including oxidative stress, mitochondrial dysfunction, inflammatory signaling, endothelial dysfunction, and G1 cell cycle arrest, without detectable changes in conventional functional indices. Biomarkers such as TIMP-2·IGFBP7 emerge at this stage, reflecting stress-adaptive and potentially reversible injury states. As injury progresses, structural damage to renal tubular cells becomes evident, accompanied by the release of biomarkers associated with tubular injury and inflammation, including NGAL, KIM-1, IL-18, L-FABP, cystatin C, and suPAR. In the later phase, overt functional deterioration manifests as reduced urine output and rising serum creatinine levels, which remain the basis of current diagnostic criteria. This biological-functional dissociation highlights the temporal gap between early kidney injury and conventional functional diagnosis, underscoring the value of mechanism-based biomarkers for early risk identification and timely clinical intervention.

Early detection of AKI is shifting from reliance on isolated functional monitoring toward mechanism-driven risk stratification. Although serum creatinine and urine output remain the diagnostic cornerstones, they primarily reflect functional changes and fail to capture early molecular events (26, 27). Advances in basic and translational research have revealed that a range of biomarkers generate stage-specific signals across different pathological phases (Figure 2), encompassing structural injury, cellular stress, and inflammatory responses. Classifying these biomarkers according to their underlying mechanisms and integrating them with functional indicators may enhance the early detection and risk stratification of AKI (Table 1).

**Table 1:** Classification, sources, and clinical implications of AKI biomarkers across distinct mechanistic dimensions.

Biomarker	Primary cellular source	Core pathophysiological process	Sample type	Temporal appearance (relative to serum creatinine)	Principal clinical implications
NGAL (12, 20, 22, 35)	Distal tubular and collecting duct epithelial cells	Tubular structural injury and epithelial dedifferentiation	Blood/Urine	Markedly earlier (hours to 24 h)	Early detection of tubular injury, differentiation between pre-renal and intrinsic AKI, prediction of AKI onset and adverse outcomes
KIM-1 (19, 40)	Proximal tubular epithelial cells	Epithelial cell detachment, repair, and regeneration	Urine	Earlier (approximately 12–24 h)	Indicator of proximal tubular injury, associated with AKI severity and duration
NAG (39, 131)	Lysosomes of proximal tubular epithelial cells	Brush border injury and lysosomal dysfunction	Urine	Earlier	Reflects impaired proximal tubular reabsorptive function, mainly used for disease course assessment
β₂-microglobulin (40, 105, 129)	Proximal tubular epithelial cells	Tubular reabsorptive dysfunction	Urine	Earlier	Adjunctive marker of functional tubular injury
TIMP-2 (13, 33, 42, 46, 47, 52)	Tubular epithelial cells	Cellular stress and G1-phase cell-cycle arrest	Urine	Markedly earlier (hours)	Identification of reversible stress states, prediction of moderate-to-severe AKI risk
IGFBP7 (13, 33, 42, 46, 47, 52)	Tubular epithelial cells	Cell-cycle arrest and metabolic suppression	Urine	Markedly earlier (hours)	Used in combination with TIMP-2 for risk enrichment and intervention triggering
IL-18 (14, 64)	Immune cells and injured tubular cells	Inflammatory activation and immune-mediated injury	Blood/Urine	Earlier	Identification of inflammation-driven AKI, particularly sepsis-associated AKI
sTNFR1/sTNFR2 (67, 132)	Immune cells	Activation of TNF-related inflammatory pathways	Blood	Earlier	Prediction of AKI development and mortality risk, reflection of systemic inflammatory burden
suPAR (71, 72)	Immune cells	Chronic immune activation and vulnerability phenotype	Blood	Baseline or early elevation	Risk enrichment marker predicti
L-FABP (15, 133)	Proximal tubular epithelial cells	lipid metabolic and mitochondrial dysfunction	Urine	Earlier	Reflecting a hypoxia- and energy depletion–associated AKI phenotype
GDF-15 (39, 76, 77)	Diverse stress-responsive cells	Metabolic reprogramming and stress responses	Blood/Urine	Earlier	Indicating systemic stress burden and adverse prognosis
8-OHdG (79, 134)	injured tubular epithelial cells	Oxidative DNA damage	Blood/Urine	Earlier	Reflecting the severity of oxidative stress and the extent of cellular injury
Cystatin C (81–83)	Nucleated cells	Non-structural alterations in glomerular filtration function	Blood	Preceding serum creatinine elevation	Enabling early assessment of GFR decline and prediction of AKI progression

AKI, acute kidney injury; Scr, serum creatinine; UO, urine output; RRT, renal replacement therapy; KDIGO, Kidney Disease: Improving Global Outcomes; NGAL, neutrophil gelatinase-associated lipocalin; KIM-1, kidney injury molecule-1; NAG, N-acetyl-β-D-glucosaminidase; β₂-MG, β₂-microglobulin; TIMP-2, tissue inhibitor of metalloproteinases-2; IGFBP7, insulin-like growth factor-binding protein 7; IL-18, interleukin-18; TNF, tumour necrosis factor; sTNFR1/2, soluble tumour necrosis factor receptor 1/2; suPAR, soluble urokinase plasminogen activator receptor; L-FABP, liver-type fatty acid-binding protein; GDF-15, growth differentiation factor 15; 8-OHdG, 8-hydroxy-2′-deoxyguanosine; GFR, glomerular filtration rate; CysC, cystatin C; SA-AKI, sepsis-associated acute kidney injury; AI, artificial intelligence; HRV, heart rate variability; RRI, renal resistive index; VExUS, venous excess ultrasound score; ROS, reactive oxygen species; DAMPs, damage-associated molecular patterns.

### 4.1 Traditional functional biomarkers: from delayed diagnosis to dynamic monitoring

#### 4.1.1 Established role and intrinsic limitations

Serum creatinine and urine output have long formed the foundation of diagnostic and staging systems for AKI, as they reflect overall changes in glomerular filtration function (1). However, these functional indicators typically become abnormal only 48–72 h after structural injury, limiting their utility for early detection. Moreover, serum creatinine is readily influenced by non-renal factors, and urine output lacks specificity, complicating the differentiation of reversible hemodynamic alterations from intrinsic renal parenchymal injury (28, 29). Although urine output provides relatively real-time information, its specificity remains limited and is susceptible to confounding by volume status, diuretic administration, and circulatory conditions (30). Urinary protein and specific gravity may partially reflect tubular reabsorptive function and osmotic regulation but are mainly used for disease monitoring rather than early injury identification (31, 32).

#### 4.1.2 Functional-structural dissociation and subclinical AKI

The concept of functional-structural dissociation highlights that changes in conventional functional indicators lag behind cellular and molecular injury processes. Even when serum creatinine and urine output remain within normal ranges, tubular stress responses and structural injury pathways may already be activated, indicating a state of subclinical AKI (33, 34). This stage is characterized by a high degree of reversibility and represents a critical window for precise intervention (35, 36). With advances in monitoring technologies and data analytics, the use of traditional indicators is shifting from static measurement to dynamic trend assessment. For example, continuous urine output monitoring combined with machine learning algorithms has been applied to identify risk trajectories several hours before AKI onset, enabling earlier warning and risk stratification (37, 38).

### 4.2 Tubular injury cluster: early signals of structural damage

#### 4.2.1 Tubular injury marker cluster

Tubular injury represents one of the earliest structural alterations in AKI, and its associated molecular biomarkers can be detected before functional indicators become abnormal, forming the “tubular injury biomarker cluster” (39). Among these, NGAL and KIM-1 are the most widely used early indicators of structural damage, while N-acetyl-*β*-D-glucosaminidase (NAG) and β₂-microglobulin primarily reflect impaired proximal tubular reabsorptive function (39–42).

#### 4.2.2 Clinical evidence and subclinical AKI detection

Recent clinical studies have validated the early warning value of this biomarker cluster (43, 44). A multicenter prospective study involving 159 patients demonstrated significantly higher urinary NGAL and KIM-1 levels in individuals with AKI than in those without. Urinary NGAL showed moderate discriminatory performance, with an area under the receiver operating characteristic curve (AUC) of 0.71 (95% confidence interval [CI] 0.57–0.86), corresponding to a sensitivity of 55% and a specificity of 89%. Urinary KIM-1 demonstrated slightly superior performance, with an AUC of 0.75 (95% CI 0.63–0.86), and a sensitivity and specificity of 80 and 65%, respectively (43). Furthermore, Haase et al. analyzed ten multicenter prospective cohorts, including 2,322 critically ill patients, and reported consistent findings across studies and meta-analyses. Elevated NGAL levels identified high-risk patients whose serum creatinine remained within normal ranges and were strongly associated with increased need for RRT (2.5% vs. 0.15%, odds ratio [OR] 16.4, 95% confidence interval [CI] 3.6–76.9) and adverse clinical outcomes, including higher in-hospital mortality (12.4% vs. 4.8%) and longer ICU length of stay (7.1 days vs. 4.2 days) (35).

In prospective perioperative cohorts, urinary [TIMP-2]•[IGFBP7] demonstrated excellent early predictive performance for postoperative AKI. At 2 h after ICU admission, it achieved an AUC of 0.932 (95% CI 0.835–1.000), corresponding to a sensitivity of 84.6% and a specificity of 96.0%, outperforming most single structural injury biomarkers (45). Thus, structural biomarkers are can identify the subclinical phase of AKI. Their dynamic changes reflect the temporal evolution of tubular injury and constitute a key triggering layer within early warning systems, thereby providing an essential window for precise intervention.

### 4.3 Cell stress and cell-cycle arrest cluster: a window of reversible injury

#### 4.3.1 G1-phase arrest as a reversible protective mechanism

During the early stage of AKI, tubular epithelial cells can induce G1-phase cell cycle arrest to temporarily halt proliferative activity, thereby shifting into a repair-prioritized, low-metabolic state that limits injury propagation and facilitates functional recovery (46–48). This adaptive response is considered a pivotal biological transition point from structural injury to reversible repair in AKI. During this process, injured cells release cell cycle arrest-associated molecular signals, including tissue inhibitor of TIMP-2 and IGFBP7, which constitute the molecular basis of cell cycle arrest biomarkers (49).

#### 4.3.2 Clinical predictive value and interventional plasticity of TIMP-2·IGFBP7

TIMP-2 and IGFBP7 are the most representative biomarkers of cellular stress and cell cycle arrest (33, 45). Elevated levels can be detected several hours before the clinical diagnosis of AKI, reflecting tubular cellular stress (13, 50). In a multicenter study of 375 patients, Gunnerson et al. reported that the combined biomarker predicted moderate-to-severe AKI within 12 h in high-risk surgical patients with an AUC of 0.84 (95% CI 0.76–0.90), highlighting its value for perioperative risk stratification (50). Besides, in a prospective cohort of 124 patients, urinary TIMP-2·IGFBP7 was significantly associated with RRT requirement and renal recovery. The AUC for predicting RRT was 0.73 (*p* = 0.001), corresponding to a sensitivity and a specificity of 81.2 and 57.4%, respectively (51).

Furthermore, the SAPPHIRE study reported that combined urinary TIMP-2·IGFBP7 measurement achieved an AUC of approximately 0.80 for predicting severe AKI, outperforming traditional structural biomarkers such as NGAL and KIM-1 (33). Importantly, the PREVAKI randomized controlled trial demonstrated that using this biomarker as a trigger for implementing KDIGO renal-protective strategies significantly reduced the incidence of postoperative AKI following cardiac surgery (55.1% vs. 71.7%, absolute risk reduction 16.6%, *p* = 0.004), indicating that this biomarker signal possesses both predictive and interventional capacity (52). At a biological level, TIMP-2·IGFBP7-related pathways reflect the dynamic balance between cell cycle arrest and stress repair, with expression levels distinguishing adaptive repair from ongoing injury (53). Accordingly, this biomarker is regarded as an early warning signal for AKI and a molecular indicator of the injury-repair inflection point. Dynamic monitoring may, therefore, help identify reversible injury windows and guide precision intervention.

#### 4.3.3 Limitations

Despite demonstrated predictive performance and interventional potential in both experimental and clinical studies, limitations persist in the clinical application of TIMP-2·IGFBP7. First, this biomarker primarily reflects tubular cellular stress rather than irreversible structural injury; elevated levels do not necessarily indicate progression to clinically overt AKI. In settings of systemic stress or hemodynamic fluctuations, urinary TIMP-2·IGFBP7 levels may transiently increase, raising the risk of false-positive interpretation in populations with low pre-test probability (54, 55).

Currently recommended cut-off values of 0.3 and 2.0 (ng/mL)^2^/1000 were derived from predictive studies conducted in specific high-risk populations, such as patients with sepsis or those undergoing cardiac surgery (56, 57). These thresholds primarily intended for short-term risk stratification of severe AKI, and their generalizability across different baseline renal function levels or etiological contexts remains uncertain. Although existing studies have statistically adjusted for clinical factors such as chronic kidney disease (CKD), optimization of stratified thresholds based on baseline renal function remains to be established. Consequently, the applicability of these cut-offs across varying estimated glomerular filtration rate strata or differing etiological backgrounds requires further prospective validation (58). Therefore, TIMP-2·IGFBP7 is best positioned as an adjunctive signal for risk stratification and management intensification rather than as a substitute diagnostic criterion. Its principal value lies in optimizing the timing of intervention rather than independently determining therapeutic pathways.

### 4.4 Inflammatory and immune response cluster: core pathways in sepsis-associated AKI

An imbalance of inflammatory and immune responses plays a central role in the development and progression of sepsis-associated acute kidney injury (SA-AKI) (59). Unlike AKI primarily driven by hemodynamic abnormalities or prerenal factors, SA-AKI is characterized by sustained activation of systemic inflammatory signaling, dysregulated immune modulation, and aberrant interactions between tubular epithelial cells and immune cells (60–62). Multiple circulating molecules reflecting inflammatory burden and immune activation have demonstrated strong associations with early AKI onset and adverse clinical outcomes.

#### 4.4.1 IL-18

IL-18, one of the most extensively studied representative molecules within this cluster, is produced by both activated immune cells and injured tubular epithelial cells (16). As a pro-inflammatory cytokine, IL-18 enhances interferon-*γ* (IFN-γ) production, amplifying local inflammatory responses and promoting a pro-inflammatory renal microenvironment, exacerbating tubular injury (63, 64). Multiple cohort studies have demonstrated that serum or urinary IL-18 levels increase significantly before elevations in serum creatinine and are positively associated with AKI severity and mortality risk (64–66). In a cohort of patients with severe sepsis, serum IL-18 levels were markedly elevated prior to AKI diagnosis (7.53 ± 6.17 vs. 2.85 ± 1.56 ng/L, *p* = 0.001), with good predictive performance for AKI within the first 24 h of admission (AUC = 0.861, 95% CI 0.778–0.944; sensitivity 66.7%, specificity 95.1%), indicating strong early warning value (66). Similarly, in a prospective study of 55 patients undergoing cardiopulmonary bypass cardiac surgery, urinary IL-18 levels increased significantly within 4–6 h postoperatively in patients who developed AKI, peaking at more than 25-fold within 12 h, whereas serum creatinine typically increased only after 48–72 h. The AUC for AKI prediction at 12 h was 0.75, further supporting its early predictive utility (64).

#### 4.4.2 Soluble tumor necrosis factor receptors (sTNFR)

Among circulating biomarkers associated with immune-mediated AKI, sTNFR1/2 have demonstrated high predictive value. Clinical studies indicate that elevated sTNFR1/2 levels can predict AKI occurrence before significant changes in serum creatinine, with AUC values for moderate-to-severe AKI generally ranging from 0.75 to 0.85 and they are strongly associated with mortality risk (67–69). A prospective cohort study involving 301 ICU patients with acute respiratory illness showed that each two-fold increase in sTNFR1 levels was associated with a 56% higher risk of severe AKI (adjusted relative risk [aRR] 1.56, 95% CI 1.24–1.96), supporting its role as an early predictor of immune activation–related AKI (68).

#### 4.4.3 Soluble urokinase plasminogen activator receptor (suPAR)

suPAR emerges as another immune activation-related biomarker of AKI. Elevated baseline suPAR levels have been associated with a two- to four-fold increase in AKI risk, clearly demonstrating a dose–response relationship with the need for RRT and adverse outcomes (70–72). In a study of 1,709 patients undergoing allogeneic hematopoietic stem cell transplantation, elevated suPAR levels on day 7 post-transplant predicted dialysis-requiring AKI (AUC 0.75) and were significantly associated with reduced overall survival, highlighting its value in early prediction and prognostic assessment (70). Moreover, a prospective study reported that combined measurement of suPAR and sTNFR2 significantly improved prediction), which exceeded that of either marker alone (67).

#### 4.4.4 Potential etiological specificity of inflammatory biomarkers in AKI

Although no prospective studies have directly compared the discriminative performance of sTNFR and suPAR across differing AKI etiologies, existing evidence suggests that they may reflect distinct inflammation-driven pathways.

##### 4.4.4.1 sTNFR serves as an indicator of structural injury burden

sTNFR1/2 appear to preferentially reflect the burden of renal parenchymal structural injury. Renal biopsy studies have shown that sTNFR1 and sTNFR2 levels are independently associated with structural lesions, including acute tubular injury, interstitial fibrosis, and tubular atrophy, and mesangial expansion. These associations persist after adjustment for baseline eGFR, indicating that sTNFR primarily reflects structural injury burden and chronic remodeling processes. In contrast, suPAR is more closely related to baseline renal function and shows weaker associations with histopathological progression in kidney disease (73). Elevated sTNFR levels are more commonly observed in AKI accompanied by inflammatory parenchymal injury, whereas their changes may be less pronounced in predominantly functional or transient hemodynamic AKI.

##### 4.4.4.2 suPAR drivers immune inflammation and hemodynamic regulation

In contrast, suPAR directly participates in immune cell activation and endothelial function regulation. A prospective study of 200 patients with sepsis demonstrated that suPAR levels consistently distinguished different severity stages of sepsis-induced AKI early in the disease course and outperformed several traditional renal injury biomarkers in predicting RRT requirements and mortality (62). Importantly, translational research indicates that suPAR can induce kidney-specific vasoconstriction by modulating calcium signaling in mesangial cells and afferent arterioles, thereby reducing renal blood flow; these hemodynamic alterations may precede overt structural injury (74).

Taken together, unlike sTNFR, which primarily reflects structural injury burden, suPAR integrates both immune inflammatory activation and hemodynamic regulatory effects. Combined assessment of these biomarkers may therefore provide a more comprehensive characterization of inflammation-driven AKI phenotypes; however, their practical discriminative value in etiological stratification requires further prospective validation.

### 4.5 Metabolic and oxidative stress cluster: signals of energy imbalance and medullary hypoxia

Metabolic imbalances and oxidative stress are integral to the AKI trajectory. These processes primarily arise from mitochondrial energy metabolism dysfunction in tubular epithelial cells, leading to disruption of redox homeostasis and metabolic reprogramming. They therefore serve a critical pathological link between early functional abnormalities and subsequent structural injury.

#### 4.5.1 Biomarkers of energy metabolic imbalance

L-FABP, growth differentiation factor-15 (GDF-15), and 8-hydroxy-2′-deoxyguanosine (8-OHdG) reflect AKI phenotypes associated with metabolic dysregulation and oxidative stress from distinct perspectives. L-FABP, released predominantly by proximal tubular epithelial cells, increases in response to hypoxia and impaired energy metabolism and is regarded as a robust indicator of metabolic stress-related AKI (15, 75). In a prospective cohort of 212 patients with sepsis or septic shock, urinary L-FABP measured at admission demonstrated significantly higher predictive performance for sepsis-associated AKI compared with serum creatinine, achieving an AUC of 0.94 (95% CI 0.90–0.97), with sensitivity and specificity values of 89.9 and 86.3%, respectively. Urinary L-FABP also predicted the need for RRT (AUC 0.74, sensitivity 71.4%, specificity 63.6%), supporting its potential role in early diagnosis and risk stratification of AKI (15).

GDF-15 is strongly associated with cellular metabolic reprogramming and stress responses, with its elevation reflecting adaptive regulatory processes activated under energy stress and inflammatory microenvironments (76, 77). Experimental studies have demonstrated significant upregulation of GDF-15 in lipopolysaccharide-induced sepsis models. Overexpression attenuates pulmonary endothelial inflammation, reduces levels of intercellular adhesion molecule-1, vascular cell adhesion molecule-1, TNF-*α*, and IL-6, and improves barrier repair, whereas downregulation exacerbates tissue injury (76).

#### 4.5.2 Biomarkers of oxidative stress and DNA damage

8-OHdG is a classical biomarker reflecting the extent of oxidative DNA damage. Its elevation in AKI patients indicates that oxidative stress has caused substantial injury to the genetic material of tubular epithelial cells (78). A matched case–control study found significantly elevated serum 8-OHdG levels in patients with diabetic kidney disease compared to those without renal injury (4.6 ± 0.7 vs. 4.0 ± 0.8 ng/mL, *p* = 0.002). Elevated 8-OHdG levels were independently associated with diabetic kidney disease (OR 2.90, 95% CI 1.15–7.34) and showed a significant inverse correlation with glomerular filtration rate, further supporting the pivotal role of oxidative stress in renal injury progression (79). Additionally, renal ischemia–reperfusion models indicate that AKI induces mitochondrial oxidative stress and DNA oxidation in distant organs, accompanied by ciliary structural disruption and aggravated tissue injury, indicating that oxidative stress is a key driver of systemic organ damage mediated by AKI (80).

#### 4.5.3 Biomarker of early functional filtration biomarker

Although cystatin C has traditionally been used to assess glomerular filtration function, accumulating evidence indicates that its levels increase before or at the very early stages of AKI, with temporal changes occurring earlier than serum creatinine (81–83). A multicenter cohort study involving 52,333 hospitalized neonates found that diagnostic criteria based on cystatin C identified approximately 6.5 times more AKI cases than conventional KDIGO creatinine-based criteria and significantly improved detection of high-risk mortality populations. Notably, AKI defined solely by cystatin C remained independently associated with higher in-hospital mortality risk (hazard ratio [HR] 2.86, 95% CI 2.02–4.04), suggesting superior early diagnostic and risk stratification performance (82). In critically ill, septic, and perioperative populations, admission or early postoperative cystatin C levels are also strongly associated with subsequent AKI occurrence and progression, effectively identifying patients likely to develop moderate-to-severe AKI (83, 84). Further studies have shown that the incidence of subclinical AKI defined by urinary cystatin C is approximately 15.6% and is significantly associated with higher 30-day mortality risk (HR 2.42); the worst outcomes was observed in patients classified as AKI substage B with positive urinary cystatin C (HR 2.83), highlighting its value in early stratification and severity assessment (85).

Overall, this biomarker cluster reflects a critical transition stage in AKI from reversible functional impairment to structural injury, providing valuable information for early risk stratification and disease course monitoring.

### 4.6 Multi-omics and composite panels

With the rapid development of high-throughput omics technologies, AKI biomarker research is shifting from the exploration of individual molecules toward a systems-level framework based on multidimensional data integration (86).

Multi-omics analyses have revealed dynamic changes in molecular networks throughout AKI pathogenesis, demonstrating substantial heterogeneity across different etiologies regarding inflammatory responses, metabolic dysregulation, and cell fate regulation. For instance, in SA-AKI, integrating single-cell and spatial transcriptomics enables the precise tracing of the cellular origins of urinary exosomal biomarkers, improving their specificity for renal cellular injury (87).

Proteomic and metabolomic studies have characterized the molecular landscape of sepsis-related AKI, indicating marked activation of immune-related signaling pathways in the kidney during infection alongside mitochondrial dysfunction. These findings suggest that immune inflammation and energy metabolic impairment are crucial molecular drivers of this condition (88). Through integrating biomarkers across different mechanistic domains into composite panels and combining them with machine learning algorithms, predictive models can be constructed to achieve comprehensive AKI risk assessment within a unified framework. Compared with single biomarkers, such approaches demonstrate superior predictive performance and more stable risk stratification capability (89). For instance, a study used urinary metabolomic data from pediatric AKI patients to develop a machine learning classification model to identify high-risk individuals during the pre-AKI stage (before the occurrence of functional injury) and achieved an AUC of 0.93 (95%CI 0.85–1.0) (90).

## 5 Dynamic and AI-enhanced early warning systems

**Figure 3:**
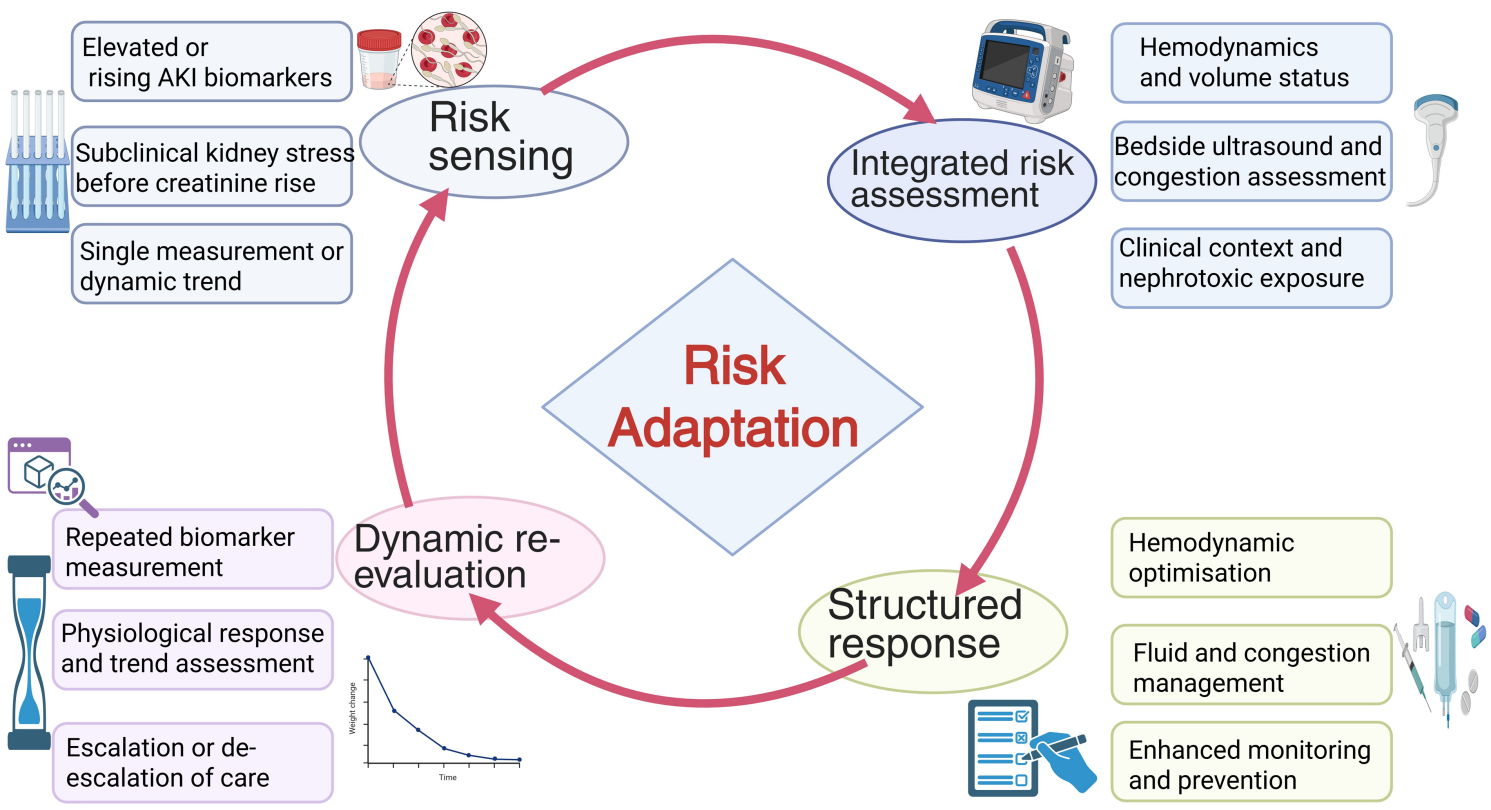
Biomarker-guided adaptive response loop for early acute kidney injury. A biomarker-guided early warning and response loop for AKI. Elevated or rising AKI biomarkers trigger early risk sensing, identifying subclinical kidney stress before overt changes in serum creatinine or urine output. This signal prompts an integrated risk assessment that contextualizes biomarker information with hemodynamic status, volume balance, bedside ultrasound findings, and clinical exposures such as sepsis or nephrotoxins. Based on this comprehensive evaluation, a structured response is initiated, focusing on hemodynamic optimization, fluid and congestion management, avoidance of nephrotoxic insults, and enhanced monitoring. Dynamic re-evaluation through repeated biomarker measurements and assessment of physiological trends enables timely escalation or de-escalation of care, forming a continuous adaptive decision loop that supports earlier and more individualized AKI management.

With the growing understanding of AKI pathogenesis, early warning research is shifting from static measurements to dynamic risk assessment frameworks that incorporate temporal information, and these approaches are increasingly being validated in real-world clinical settings (Figure 3).

### 5.1 Dynamic trajectories of biomarkers

Continuous monitoring of biomarker dynamics equips vital insights into the temporal evolution of AKI (91, 92). Experimental and clinical studies indicate that circulating TNFR1/2 levels rise significantly within one hour after kidney injury, and their levels correlating positively with injury severity, whereas traditional functional indicators such as serum creatinine exhibit delayed changes (93, 94). This temporal discrepancy reflects stage-specific differences between early molecular activation and later functional deterioration. Furthermore, in critically ill AKI patients requiring CRRT, dynamic trajectories of cTNFR1 yield greater prognostic value compared to single-point measurements. Patients with the highest tertile increase from day 0 to day 2 had significantly higher mortality risk (*p* = 0.033), with baseline cTNFR1 levels independently predicting in-hospital mortality (HR 1.82, 95% CI 1.09–3.03) and progression from AKI to CKD (*p* = 0.014) (95).

In clinical applications, biomarkers such as NGAL and TIMP-2·IGFBP7 are widely used to characterize the dynamic features of tubular stress and injury (96). Rapid short-term fluctuations in these biomarkers indicate ongoing renal stress or progressive injury. Even when absolute levels remain below conventional diagnostic thresholds, such changes are closely associated with subsequent AKI development and adverse outcomes (50). In contrast, single time-point measurements are more susceptible to baseline variability and non-specific fluctuations, potentially underestimating true risk.

### 5.2 Artificial intelligence-based decision support for AKI early warning

Integrating longitudinal biomarker monitoring and time-series clinical data has facilitated the application of artificial intelligence (AI)-based computational approaches to include multi-time-point and multivariable information for dynamic AKI risk assessment and decision support (89, 97, 98).

Models using long short-term memory deep learning architectures have integrated time-series data, including serum creatinine trajectories, urine output, and hemodynamic parameters, applying attention mechanisms to enhance identification of critical time windows. These models demonstrate higher accuracy and earlier detection capabilities for AKI prediction compared with traditional methods (99). Such models can identify patients with persistently increasing risk before overt changes in serum creatinine, allowing earlier stratification of individuals likely to develop AKI or progress to moderate-to-severe stages (100).

Dynamic prediction models based on time-dependent feature modeling can continuously update risk estimates throughout the disease course, thereby providing time-sensitive clinical decision support. The multicenter ICU-based deep learning model ORAKLE has demonstrated superior performance compared with conventional statistical and standard machine learning approaches in predicting 30-day major adverse kidney events in AKI patients (97). However, the clinical implementation of these models still depends on their interpretability and the stability of external validation results to ensure that outputs remain comprehensible to clinicians and applicable in practice (101).

### 5.3 Real-world validation and interpretability

The clinical value of dynamic AKI early warning models depends on their robustness and generalizability in real-world settings. Given the substantial heterogeneity in real-world data concerning patient populations, disease spectra, and monitoring frequency, model generalizability remains a major challenge (102). Consequently, multicenter external validation are crucial in evaluating clinical applicability.

In recent years, predictive models developed using large-scale ICU databases have undergone repeated validation across diverse patient populations, assessing discrimination, calibration performance, and risk stratification consistency to reduce the risks of overfitting and context-specific bias (37, 103). Meanwhile, interpretability analyses that quantify the contributions of variables and their temporal dynamics to risk prediction improve model transparency and facilitate integration with clinical decision-making (104). Overall, real-world validation and interpretability analyses are essential prerequisites for the clinical translation of dynamic AKI early warning models.

## 6 Biomarker-guided clinical response pathways

The clinical significance of AKI biomarkers extends beyond risk prediction to their potential linkage with specific therapeutic interventions that can modify disease progression. Accordingly, incorporating early warning signals into structured response pathways represents a critical step toward their clinical translation. Triggering interventions based on predefined thresholds or dynamic changes, combined with continuous reassessment to establish a closed-loop management process, may facilitate effective alignment between risk identification and treatment decision-making. Table 2 summarizes representative clinical evidence supporting the role of these biomarkers in risk stratification and early intervention.

**Table 2:** Clinical evidence and actionable implications of kidney injury biomarkers for AKI risk stratification and early intervention.

Biomarker	Study	Study type	Population	Cohort structure	Sample size	Detection method	AKI-related outcomes	Key findings	Recommended clinical actions
NGAL	Törnblom S, 2020, *Ann Intensive Care* (12)	Prospective multicentre cohort study	ICU patients with sepsis (FINNAKI cohort)	Single cohort	484	ELISA	KDIGO AKI, RRT, mortality	Urinary NGAL showed moderate discrimination for AKI, severe AKI, and RRT; however, decision curve analysis demonstrated minimal net clinical benefit, limiting its utility as a stand-alone predictive or decision tool in septic ICU patients	Use as a structural injury–weighted risk marker to increase clinical vigilance and monitoring intensity; not recommended as a sole trigger for intervention
NGAL	Gambino C, 2023, *Hepatology* (41)	Retrospective observational cohort study	Cirrhosis patients	Single cohort	162	Chemiluminescent immunoassay	AKI phenotypes, mortality	Urinary NGAL levels were significantly higher in ATN-AKI than in prerenal AKI or HRS-AKI; uNGAL ≥220 ng/mL predicted non-response to terlipressin plus albumin and independently associated with in-hospital mortality	Support AKI aetiological differentiation and treatment response prediction; assist in distinguishing structural injury from functional AKI and guiding therapy and prognostic assessment
suPAR	Hayek SS, 2020, *N Engl J Med* (71)	Multicentre prospective observational cohort with translational experimental validation	Hospitalized adults undergoing coronary angiography, cardiac surgery, or ICU admission	Multiple independent clinical cohorts	3,827	ELISA	Incident AKI, AKI or death	Baseline suPAR levels demonstrated a dose–response relationship with AKI risk and identified high-risk individuals before serum creatinine elevation	Use as a susceptibility marker for early risk enrichment, enabling upstream preventive strategies (contrast exposure, perioperative planning, nephrotoxin stewardship)
TIMP-2·IGFBP7	Greco M, 2023, *Diagnostics* (46)	Prospective observational cohort study	Critically ill COVID-19 patients admitted to ICU	Single-centre prospective cohort	41	Fluorescence immunoassay	KDIGO AKI	Urinary [TIMP-2]·[IGFBP7] increased only at 12 h after ICU admission in patients who developed severe AKI, suggesting a narrow and context-dependent detection window	Use to signal short-term AKI risk within defined temporal windows; interpret in conjunction with clinical context and complementary indicators
TIMP-2·IGFBP7	Yang HS, 2022, *Ann Lab Med* (13)	Prospective multicentre observational cohort study	High-risk emergency department patients	Single-centre prospective cohort	529	Fluorescence immunoassay	KDIGO AKI	ED admission [TIMP-2]·[IGFBP7] outperformed clinical ED scores in AKI prediction, improved risk reclassification, and associated with 30-day mortality	Support early rule-in and rule-out of AKI risk in the ED, enabling admission-level stratification and preventive management
TIMP-2·IGFBP7	Jia HM, 2022, *Ann Intensive Care* (47)	Prospective observational cohort study with derivation and validation phases	Adult ICU AKI patients	Two-phase design: derivation cohort(n = 194) and validation cohort (n = 185)	379	Fluorescence immunoassay	AKI recovery versus progression	Day 0 urinary [TIMP-2]·[IGFBP7] predicted non-recovery from AKI; predictive performance improved significantly when combined with clinical variables	Differentiate reversible from progressive AKI; inform early escalation of care and timely nephrology consultation
IL-18	Kuo P-Y, 2023, *Biomolecules* (14)	Single-centre prospective observational cohort study	Patients undergoing cardiac catheterisation	Single cohort with serial biomarker measurements at three time points	94	ELISA	AKI and AKD	Post-procedural AKD incidence was high; sustained elevation of urinary IL-18 and its early dynamics independently predicted subsequent AKD	Identify risk of persistent kidney injury after AKI; support extension of management beyond the acute phase into recovery and follow-up
Cystatin C	Haines RW, 2023, *CJASN* (29)	Single-centre prospective longitudinal observational cohort study	Mechanically ventilated critically ill ICU patients with prolonged stay	Single cohort with repeated longitudinal measurements and gold-standard GFR validation	38	Turbidimetric immunoassay	Bias in kidney function assessment, persistent dysfunction	Cystatin C more accurately reflected true GFR than serum creatinine, avoiding systematic overestimation related to muscle loss	Detect persistent kidney dysfunction or AKD after AKI; avoid sole reliance on creatinine-based assessment
Cystatin C	Chen J, 2023, *Ann Intensive Care* (85)	Prospective multicentre observational cohort study	Critically ill children admitted to PICUs in four tertiary hospitals in China	Single prospective cohort without internal discovery–validation split	793	Turbidimetric immunoassay	KDIGO AKI plus uCysC-based substages	Sub-AKI defined by uCysC ≥1.26 mg/g uCr identified KDIGO-negative patients with high mortality risk	Enable refined AKI risk stratification and identification of subclinical AKI; guide monitoring intensity and management escalation

AKI, acute kidney injury; AKD, acute kidney disease; ATN, acute tubular necrosis; HRS-AKI, hepatorenal syndrome–acute kidney injury; ICU, intensive care unit; ED, emergency department; PICU, paediatric intensive care unit; KDIGO, Kidney Disease: Improving Global Outcomes; RRT, renal replacement therapy; NGAL, neutrophil gelatinase-associated lipocalin; suPAR, soluble urokinase plasminogen activator receptor; TIMP-2, tissue inhibitor of metalloproteinases-2; IGFBP7, insulin-like growth factor-binding protein 7; IL-18, interleukin-18; CysC, cystatin C; uCysC, urinary cystatin C; GFR, glomerular filtration rate; AUC, area under the receiver operating characteristic curve; HR, hazard ratio; OR, odds ratio; CI, confidence interval.

### 6.1 Biomarker-triggered KDIGO bundle

#### 6.1.1 Trigger logic: operationalizing biomarker signals

Biomarker-driven clinical pathways for AKI are not centered on the biomarkers themselves but on how biological signals are translated into actionable decision triggers. Based on prior studies and interventional trial experiences, triggering strategies can be broadly classified into three complementary modes: threshold-based triggering, dynamic trend triggering, and composite risk triggering (20, 50, 105). Threshold-based triggering relies on previously validated cutoff values to identify patients who have already entered a high-risk state. Trend-based triggering focuses on the magnitude and direction of biomarker changes over time, thereby capturing individuals whose risk is rapidly accumulating despite not yet crossing fixed thresholds. Composite triggering further integrates biomarker signals with specific high-risk clinical exposures, which enhances risk identification specificity in complex critical care settings.

The shared objective of these triggering strategies is to initiate intervention earlier during the subclinical stage of tubular stress. This phase typically precedes rises in serum creatinine and declines in urine output, characterized predominantly by functional and potentially reversible changes. Therefore, the first 0 to 6 h following trigger activation are regarded as a critical intervention window. Notably, a negative biomarker result does not completely exclude the risk of AKI. Even in large multicenter studies, discriminatory performance has remained moderate, with an AUC of approximately 0.75, yielding roughly 50% sensitivity and 80% specificity, and detection ability is particularly limited for mild AKI (106). In high-risk scenarios, such as severe hypotension, hypovolemia, or persistent inflammatory exposure, the absence of threshold crossing should not delay the initiation of interventions. Biomarkers should be regarded as risk amplifiers rather than substitutes for clinical judgment.

#### 6.1.2 Modularized implementation of the KDIGO bundle

Following the emergence of a triggering signal, the KDIGO preventive recommendations, traditionally presented as principle-based guidance, should be restructured into modular and executable intervention units to align with the fast-paced environment of critical care practice (9). This bundle does not represent a single intervention but comprises multiple interrelated functional modules. Its central rationale is the simultaneous optimization of renal perfusion, limitation of secondary injury, and strengthening of early monitoring.

Specifically, the volume and fluid management module aims to maintain a dynamic balance between hypoperfusion and fluid overload (107). The hemodynamic module emphasizes the establishment of individualized perfusion targets based on patient-specific background factors, with repeated recalibration to ensure adequate perfusion of vital organs (108). The nephrotoxic exposure management module focuses on the systematic discontinuation or substitution of potentially harmful medications to reduce additional renal injury (109). The metabolic homeostasis module addresses the stabilization of glycemic control, electrolyte balance, and acid–base status. Finally, the monitoring and reassessment module provides feedback for the aforementioned interventions and supports subsequent adjustments in clinical decision-making.

#### 6.1.3 Early RRT assessment as part of response escalation

Within biomarker-triggered clinical response pathways, decisions regarding RRT should be regarded as part of risk stratification and treatment preparedness, rather than as an immediate instruction to initiate therapy. For patients who remain hemodynamically or metabolically unstable despite early interventions, early entry into an RRT assessment phase helps to identify risks of decompensation, such as persistent fluid imbalance, metabolic disturbance, or progressive fluid accumulation (9). The focus at this stage is to evaluate the likelihood of clinical progression and to complete preparatory steps, including vascular access planning, anticoagulation strategy selection, and therapeutic communication, thereby avoiding reactive or delayed initiation when deterioration occurs.

In this context, prior studies support biomarker-driven alert response strategies. A randomized controlled trial involving 121 high-risk abdominal surgery patients found that the implementation of a KDIGO care bundle triggered by TIMP-2·IGFBP7 > 0.3 significantly reduced the incidence of moderate-to-severe AKI (*p* = 0.04), decreased the proportion of patients with postoperative creatinine increases greater than 25% (*p* = 0.01), and shortened both ICU and hospital length of stay (p = 0.04). These findings indicate that biomarker-triggered structured interventions can attenuate AKI severity and improve perioperative outcomes (110, 111). Additionally, among patients exhibiting persistent high-risk features, structured interventions guided by clinical decision support systems reduce AKI incidence, highlighting the potential value of timely response following risk identification (112, 113). Collectively, these studies provide a foundation for integrating biomarkers into clinical decision-making pathways and promote their transition from single intervention evaluation toward real-world implementation. Following risk identification, the key challenge lies in translating alert signals into individualized intervention strategies.

### 6.2 Individualized hemodynamic optimization

Critically ill patients exhibit substantial heterogeneity in cardiac function, volume status, venous return, and microcirculatory perfusion. A single blood pressure or volume target is therefore insufficient to simultaneously ensure renal perfusion while limiting venous congestion (114). Accordingly, individualized hemodynamic optimization has become a key component of biomarker-driven AKI response pathways. Its central objective is to improve effective perfusion while avoiding secondary venous congestion and volume-related injury.

#### 6.2.1 Dual determinants of renal perfusion: forward flow and backward pressure

Renal perfusion is not determined solely by systemic arterial pressure or cardiac output; it reflects the combined effects of forward perfusion adequacy and backward venous pressure load (114). Impaired forward perfusion is commonly observed in states of low mean arterial pressure (MAP), reduced cardiac output, or microcirculatory dysfunction, leading to diminished renal blood flow and reduced filtration pressure. In contrast, elevated backward pressure is primarily associated with renal venous congestion, increased intra-abdominal pressure, right ventricular dysfunction, and fluid overload. Its impact on renal function is often underestimated. In a prospective multicenter study including 125 critically ill patients with KDIGO stage 2 or higher AKI, the venous excess ultrasound (VExUS) score, constructed using Doppler signals from the portal, hepatic, and intrarenal veins, was independently associated with 30-day mortality (grade 2 adjusted hazard ratio 4.03; grade 3 adjusted hazard ratio 2.70; *p* = 0.03). These findings indicate that venous congestion is significantly associated with AKI occurrence and progression, and this relationship remains independent of conventional macrohemodynamic parameters such as MAP (115).

Based on this perfusion concept, strategies that rely solely on fluid administration or vasopressor therapy to improve forward perfusion may exacerbate venous congestion, thereby offsetting potential renal benefits. This understanding has driven increasing attention toward refined hemodynamic management and systematic monitoring of the venous circulation.

#### 6.2.2 Decision value of multimodal monitoring within the response pathway

##### 6.2.2.1 Renal perfusion and venous congestion related indicators

During the management phase following biomarker activation, the value of hemodynamic and kidney-related monitoring parameters lies not in isolated diagnosis but in guiding the direction of intervention. The renal resistive index (RRI) reflects changes in intrarenal vascular resistance. In a retrospective study of 180 patients undergoing cardiac surgery, a post-cardiopulmonary bypass RRI greater than 0.74 or 0.79 was significantly associated with postoperative AKI defined by KDIGO criteria, whereas preoperative RRI showed no such association (116). A prospective study involving 80 patients further demonstrated that preoperative RRI was independently associated with AKI occurrence (OR 2.15, 95% CI 1.07 to 4.33, *p* = 0.03). Patients with postoperative RRI greater than 0.75 had a significantly higher risk of AKI (OR 3.54, 95% CI 1.18 to 10.62, *p* = 0.02), and the combined predictive AUC across multiple time points reached 0.71 (117). These findings suggest that RRI may serve as a dynamic perioperative indicator for early detection of renal perfusion abnormalities.

Beyond forward perfusion, the VExUS scoring system, based on venous ultrasonography, provides complementary assessment of systemic venous congestion, volume load, and impaired venous return (115).

##### 6.2.2.2 Autonomic regulation and urinary dynamic indicators

Heart rate variability, as an integrated reflection of autonomic regulation and systemic stress response, has been shown to be strongly associated with circulatory instability and the risk of organ dysfunction in critically ill patients. It may help identify individuals with inadequate resuscitation response or persistent physiological stress burden (118). Meanwhile, urodynamic and urinary biochemical indices remain useful for distinguishing prerenal functional changes from intrinsic renal injury and for assessing the reversibility of dysfunction (119). Their interpretation should be integrated with fluid responsiveness assessment tools, such as the passive leg raising test and stroke volume variation, thereby enabling hemodynamic monitoring to function as a continuous decision support component within AKI response pathways (120).

#### 6.2.3 Individualized adjustment of interventions and dynamic reassessment

Based on multimodal monitoring data, intervention strategies should follow principles of individualization and staged adjustment. The use of vasoactive agents should be tailored according to patient-specific background factors such as baseline blood pressure, chronic hypertension, or renovascular disease, with differentiated perfusion targets established accordingly (121). For instance, in patients with chronic hypertension, the renal autoregulatory curve may shift rightward. Maintaining a higher MAP, typically 75 to 85 mmHg, during sepsis or hypoperfusion states may, therefore, be more conducive to preserving renal perfusion (122, 123). In a study involving patients with septic shock and a history of chronic hypertension, targeting a MAP of 80 to 85 mmHg compared with 65 to 70 mmHg significantly improved creatinine clearance, increased urine output and urinary sodium excretion, and reduced serum creatinine levels (124). The Sepsis and Mean Arterial Pressure (SEPSISPAM) trial further demonstrated that, within the chronic hypertension subgroup, a higher MAP target was associated with reduced need for RRT (*p* = 0.04) and a significantly lower incidence of doubling of serum creatinine (*p* = 0.009) (125). Additionally, perioperative randomized studies have shown that maintaining intraoperative MAP between 80 and 95 mmHg reduces the risk of AKI following major abdominal surgery (126).

Fluid management should follow the resuscitation, optimization, stabilization, and evacuation framework, transitioning from early fluid resuscitation toward restrictive and deresuscitative strategies to reduce the risk of volume-related complications (107, 127). In patients with oliguria and evidence of fluid overload, diuretic strategies should be attempted under close monitoring. When diuretic response is inadequate or metabolic burden continues to worsen, early evaluation of RRT feasibility is warranted (128). These decisions should not be made as a single event but require dynamic reassessment at predefined time points. Integrating biomarker trends, hemodynamic parameters, and urine output trajectories into joint evaluations at 6, 12, and 24 h facilitates early identification of intervention failure or risk escalation and supports subsequent therapeutic adjustment.

### 6.3 Multidisciplinary nursing and clinical coordination

Biomarker-triggered AKI response pathways do not represent isolated interventions but constitute a dynamic management process reliant on continuous information updating and multidisciplinary collaboration (111). Because AKI progression is often accompanied by rapid changes in hemodynamic status, volume load, and treatment exposures, decisions based on a single discipline or at a single time point are unlikely to reflect true risk adequately. Multidisciplinary coordination is therefore not only an operational requirement but also a structural condition necessary to maintain temporal consistency between risk assessment and intervention strategies.

Within this framework, clinical decision-making, bedside implementation, and technical support form interdependent functional units. ICU physicians are responsible for risk re-stratification and strategy adjustment. Nursing teams contribute by continuously acquiring urine output, fluid balance, and circulatory data, enabling dynamic monitoring and feedback. Early involvement of pharmacy, blood purification, and laboratory teams further assists in reducing iatrogenic injury and improving the timeliness of decision-making.

The central value of this collaborative model lies in sustaining continuity within the response pathway. By systematically reviewing intervention effects within predefined time windows and generating traceable feedback, biomarker-driven AKI alert response systems can maintain stable performance in real-world clinical settings while ensuring reproducibility and long-term sustainability.

### 6.4 Translational implementation: from research biomarkers to bedside decision

The transition of AKI biomarkers from research tools to reliable clinical decision supports is primarily constrained not by predictive performance but by the integration of detection, interpretation, and response within a coherent system. Current evidence indicates that, in the absence of clearly defined clinical contexts, standardized trigger thresholds, and corresponding intervention pathways, biomarker signals often remain limited to risk notification and fail to translate into actionable clinical measures (9). Therefore, the focus of translational implementation should shift from isolated test performance toward the establishment of a closed-loop management framework linking biomarker activation, structured response, and outcome evaluation.

At the operational level, it is first necessary to define testing time windows, reporting formats, and quality control standards to ensure inter-center comparability. Second, trigger rules should be specified in advance, linking absolute biomarker levels or dynamic changes to predefined management strategies, thereby reducing interpretative variability. During the execution phase, the priority is not the addition of new interventions but consistent implementation of established pathways, with timely reassessment when deviations occur. Finally, pathway performance should be continuously monitored through outcome indicators and dynamically refined using real-world data. This approach aligns closely with the recently proposed learning healthcare pathway framework (129, 135). Only when stable feedback loops are established between detection, decision-making, and outcome evaluation can AKI biomarkers evolve from research indicators into scalable and reproducible bedside decision tools.

## 7 Traditional challenges and future perspectives

### 7.1 Limitations of AKI biomarkers

#### 7.1.1 Biological heterogeneity and predictive uncertainty

Although novel biomarkers of kidney injury can reflect tubular stress or structural damage at a molecular level earlier than serum creatinine, their clinical application remains constrained by several limitations. First, at the biological level, most biomarkers primarily reflect cellular stress or inflammatory activation rather than a direct decline in glomerular filtration function. Consequently, their elevation does not necessarily correspond to persistent functional impairment or irreversible structural injury. Second, AKI exhibits marked pathophysiological heterogeneity. Across different etiologies and clinical contexts, similar biomarker levels may represent distinct pathological states and prognostic risks, thereby increasing uncertainty in risk stratification (1). Furthermore, although some studies have reported good discriminatory performance, the positive predictive value remains limited in populations with low AKI incidence. Most proposed thresholds are derived from specific cohorts, and the external stability of fixed cutoff values remains to be validated.

#### 7.1.2 Implementation and translational barriers

Beyond these intrinsic limitations, substantial systemic barriers remain in translating biomarkers into routine clinical practice. From a clinical decision perspective, robust evidence is still lacking to demonstrate that interventions based solely on biomarker abnormalities significantly improve long-term renal outcomes. Biomarkers can indicate the presence of risk but do not directly specify intervention pathways. Without standardized response strategies, test results are unlikely to translate into actionable clinical measures. Additionally, different biomarkers rely on heterogeneous detection technologies, including immunoassays, mass spectrometry, or point-of-care platforms. These methods vary in sensitivity, specificity, and inter-assay consistency. Sampling time windows and pre-analytical procedures are also not standardized, which limits intercenter comparability (129). Moreover, in real-world settings, factors such as testing cost, accessibility, reporting turnaround time, and integration within clinical information systems may further influence implementation efficiency. Some biomarkers are also susceptible to non-specific elevations related to inflammatory burden, volume status, and comorbid conditions.

#### 7.1.3 Health economic and implementation barriers

In addition to biological and methodological constraints, AKI biomarkers face substantial economic and implementation challenges. As shown in Table 2, most biomarkers require specialized platforms such as enzyme-linked immunosorbent assays, chemiluminescent immunoassays, or fluorescence-based detection systems. These tests are considerably more expensive than routine biochemical assays and demand advanced laboratory infrastructure and quality control, limiting their routine use in resource-constrained settings. Within healthcare systems, payers and policymakers are primarily concerned with whether biomarker-guided strategies can reduce high-cost events, such as the need for RRT, duration of dialysis, length of ICU stay, or overall hospital expenditure. From a health economic perspective, their adoption requires validation through cost-effectiveness analyses that compare biomarker-triggered intervention strategies with standard care in terms of resource utilization and clinical benefit. Without clear evidence demonstrating measurable resource savings, even biomarkers with strong predictive performance are unlikely to achieve reimbursement approval or widespread implementation.

### 7.2 Integrated and dynamic risk stratification framework

#### 7.2.1 Stage- and context-specific application of AKI biomarkers

In current clinical practice, serum creatinine and urine output remain the core functional criteria for AKI diagnosis and staging. Accordingly, any novel biomarker should be positioned as a complement rather than a replacement for functional indicators. The application of AKI biomarkers should therefore follow principles of risk context-driven and stage-specific stratification, with clearly defined functional roles.

In patients with well-defined high-risk exposures, such as major surgery, sepsis, or nephrotoxic drug use, cell cycle arrest biomarkers, particularly TIMP-2· IGFBP7, are most suitable for early risk screening. They help identify subclinical stress stages and trigger KDIGO preventive strategies (56). In patients with substantial inflammatory burden or sepsis, inflammation-related biomarkers such as IL-18, sTNFR, and suPAR can further refine risk stratification and prognostic assessment (65, 67).

For patients who already exhibit functional impairment or progressive disease, structural injury biomarkers such as NGAL and KIM-1, along with their dynamic trajectories, are more informative for assessing injury persistence and recovery potential, thereby guiding monitoring intensity or preparation for treatment escalation (20). Functional markers such as cystatin C may serve as early complements to creatinine to improve sensitivity for detecting functional decline (29). Thus, future clinical application should not focus on identifying a single optimal biomarker but rather on clarifying their respective roles in screening, stratification, and decision support across different risk stages. Biomarkers should function as stratification tools within clinical pathways rather than as isolated diagnostic tests.

#### 7.2.2 Integrated and dynamic risk stratification framework

Given that no single biomarker can fully capture the multifactorial drivers of AKI, future translational efforts should focus on establishing integrated and dynamic risk characterization frameworks. By combining molecular biomarker signals with dynamic physiological parameters, bedside imaging findings, and metabolic and inflammatory profiles, clinicians can more accurately delineate the temporal evolution of AKI risk trajectories (130).

The primary goal of such integration is not to construct increasingly complex predictive models but to better distinguish dominant pathophysiological mechanisms and thereby support differentiated and interpretable clinical response strategies. For example, when hypoperfusion, venous congestion, or tubular stress predominates, priorities for intervention and reassessment frequency should differ accordingly. By aligning risk identification with response intensity, AKI management may evolve from static risk notification toward a dynamic decision-making process centered on individualized care.

### 7.3 Future perspective

Future research should move beyond the early predictive performance of individual biomarkers toward precision management frameworks that integrate mechanistic insights with dynamic risk stratification. At the mechanistic level, multimarker strategies are needed to distinguish dominant pathological processes and, when combined with temporal trajectories, to differentiate persistent injury from reversible stress states. At the intervention level, well-designed multicenter randomized controlled trials are required to determine whether standardized response strategies triggered by biomarker abnormalities can genuinely improve renal and long-term clinical outcomes.

Additionally, artificial intelligence and multimodal data integration technologies hold promise for enhancing the precision of AKI risk stratification. However, their practical value depends on ensuring interpretability, external validity, and clinical feasibility. Translational research should also incorporate health economic evaluation to assess the impact of biomarker-guided strategies on RRT utilization, dialysis duration, ICU length of stay, and overall healthcare expenditure. Such analyses are essential to define their real-world value in resource allocation and cost control, thereby supporting future implementation and reimbursement decisions.

## 8 Conclusion

The management of AKI is shifting from a delayed diagnostic approach, based on serum creatinine and urine output, toward earlier warning and more precise responses guided by mechanism-related biomarkers. These biomarkers characterize the dynamic pathological spectrum of AKI across structural injury, cellular stress, inflammatory and immune activation, and metabolic dysregulation, thereby providing a biological foundation for identifying subclinical injury windows and initiating early intervention. However, their translational value depends on whether they can be systematically embedded within standardized, actionable, and sustainably evaluable clinical pathways that link early warning to effective response. In the future, the integration of multidimensional biological signals with continuous monitoring and decision support systems, along with ongoing validation and optimization in real-world settings, may enable AKI biomarkers to support a transition from passive recognition to proactive and individualized risk management, ultimately improving renal outcomes and overall prognosis in critically ill patients.
